# An Unstable Th Epitope of *P. falciparum* Fosters Central Memory T Cells and Anti-CS Antibody Responses

**DOI:** 10.1371/journal.pone.0100639

**Published:** 2014-07-01

**Authors:** Carlos A. Parra-López, David Bernal-Estévez, Luis Eduardo Vargas, Carolina Pulido-Calixto, Luz Mary Salazar, J. Mauricio Calvo-Calle, Lawrence J. Stern

**Affiliations:** 1 Department of Microbiology, School of Medicine, Universidad Nacional de Colombia, Bogotá, Colombia; 2 Graduate School in Biomedical Sciences, Universidad Nacional de Colombia, Bogotá, Colombia; 3 Faculty of Sciences, Universidad Nacional de Colombia, Bogotá, Colombia; 4 Fundación Salud de los Andes, Research Group of Immunology and Clinical Oncology - GIIOC, Bogotá, Colombia; 5 University of Massachusetts Medical School, Department of Pathology and Biochemistry and the Department of Molecular Pharmacology, Worcester, Massachusetts, United States of America; Queensland Institute of Medical Research, Australia

## Abstract

Malaria is transmitted by *Plasmodium*-infected anopheles mosquitoes. Widespread resistance of mosquitoes to insecticides and resistance of parasites to drugs highlight the urgent need for malaria vaccines. The most advanced malaria vaccines target sporozoites, the infective form of the parasite. A major target of the antibody response to sporozoites are the repeat epitopes of the circumsporozoite (CS) protein, which span almost one half of the protein. Antibodies to these repeats can neutralize sporozoite infectivity. Generation of protective antibody responses to the CS protein (anti-CS Ab) requires help by CD4 T cells. A CD4 T cell epitope from the CS protein designated T* was previously identified by screening T cells from volunteers immunized with irradiated *P. falciparum* sporozoites. The T* sequence spans twenty amino acids that contains multiple T cell epitopes restricted by various HLA alleles. Subunit malaria vaccines including T* are highly immunogenic in rodents, non-human primates and humans. In this study we characterized a highly conserved HLA-DRβ1*04:01 (DR4) restricted T cell epitope (QNT-5) located at the C-terminus of T*. We found that a peptide containing QNT-5 was able to elicit long-term anti-CS Ab responses and prime CD4 T cells in HLA-DR4 transgenic mice despite forming relatively unstable MHC-peptide complexes highly susceptible to HLA-DM editing. We attempted to improve the immunogenicity of QNT-5 by replacing the P1 anchor position with an optimal tyrosine residue. The modified peptide QNT-Y formed stable MHC-peptide complexes highly resistant to HLA-DM editing. Contrary to expectations, a linear peptide containing QNT-Y elicited almost 10-fold lower long-term antibody and IFN-γ responses compared to the linear peptide containing the wild type QNT-5 sequence. Some possibilities regarding why QNT-5 is more effective than QNT-Y in inducing long-term T cell and anti-CS Ab when used as vaccine are discussed.

## Introduction

Despite a significant reduction in the incidence and number deaths due to malaria, this disease claimed over 600,000 human lives in 2011 [1]. The widespread resistance of mosquitoes to insecticides and of malaria parasites to drugs greatly encourages the development of a malaria vaccine as a long-term solution. Vertebrates are infected when malaria-infected mosquitoes inoculate sporozoites during a blood meal. Studies in the last century with irradiated sporozoites [2]–[6] and more recently with cryopreserved radiation-attenuated purified *P. falciparum* sporozoites [7] demonstrate that solid and reproducible protection to malaria challenge can be achieved by immunization with sporozoites. In rodents has been shown that a major target of the protective immune response to sporozoites is the circumsporozoite (CS) protein [8] that uniformly cover the surface of the sporozoite. Studies in rodents and more recently in humans immunized with RTS,S a leading malaria vaccine based on the CS protein [9], or irradiated sporozoites have shown that anti-CS antibodies are higher in protected individuals than in those non-protected [7], [10]. In malaria mouse model protection to sporozoite challenge was achieved by passive transfer of antibodies to the CS protein [11] and in a simian host by incubation of anti-CS antibodies with sporozoites before injection in susceptible hosts [12], [13]. A major target of the anti-CS neutralizing antibodies are continuous repetitive units [13]–[16] located in the central region of the protein and that are characteristic of the malaria parasite species. In the human malaria parasite *Plasmodium falciparum* the repetitive units are represented by 6 copies of the minor (NVDP) repeats interspersed within up to 40 major (NANP) repeats. Analysis of T cell responses of individuals immunized with a synthetic vaccine [17] and of naturally exposed individuals [18] suggest that *P. falciparum* repeats are poor immunogens and antibody responses are only elicited in a restricted number of individuals of defined genotypes [19].

Responses in CD4 T cells are elicited upon engagement of T cell receptors on these cells with class II MHC (MHCII) molecules on the surface of professional antigen presenting cells (APCs). Peptides bind to MHCII molecules by a conserved network of hydrogen bonds between the peptide backbone and conserved MHCII side chains and by interactions between pockets in the MHCII binding groove and peptide side chains [20]. During assembly of MHCII-peptide complexes the peptide editor HLA-DM surveys the binding of epitopes by releasing weakly-associated peptides. This editing represents one important step in immunodominance determination. Studies on the immunogenicity of CD4 T cells for foreign viral antigens [21], model antigens [22], and antigens involved in auto-immunity [23], suggest an important role of HLA-DM in the selection of MHCII-peptide complexes with high kinetic stability. Sant and colleagues reviewed the relationship between immunodominance, HLA-DM editing, and kinetic stability of MHCII-peptide complexes and suggested that for CD4 T cell responses immunodominance is primarily due to an intrinsic property of MHC-peptide complex stability [24]. Most studies have assessed the relationship between MHCII-peptide complex stability and CD4 immunogenicity using T cell read outs such as proliferation or IL-2 and IFN-γ production [21], [22], [24], [25]. At present there is little information regarding the impact that MHC-peptide complex stability has on the capacity of CD4 T cell epitopes to provide help for production of antibodies by B cells.

In addition to antibodies, protection against *P. falciparum* sporozoites has been correlated with the presence of CD4 T helper cells producing IFN-γ in vaccinated volunteers [26] and naturally infected individuals living in Africa [27]. A good CD4 T helper epitope must be “universal” (recognized by CD4 T lymphocytes in MHC haplotypes widely distributed in the population). The search of universal epitopes in the CS protein has been successful; Sinigaglia identified a conserved region (CS.T3) recognized by different MHCII molecules in both mouse and man [28]; Doolan identified three promiscuous T cell epitopes (CSP-2, CSP-53 and CSP-375) recognized by HLA-DR molecules representative of the most common MHCII DR molecules worldwide [29] and Moreno and colleagues described an epitope named T* (CS 326–345) presented by multiple MHCII DR molecules [30]. The fine mapping of human CD4 T cell responses within universal epitopes and other regions of *P. falciparum* CS protein has identified five CD4 T helper cell epitopes designated as T1, Th2R, Th3R, CS.T3 and T* [18], [30]–[33]. The immunogenicity of Th2R, Th3R, CS.T3 and T* has been demonstrated in clinical trials by Reece and colleagues who demonstrated that response to CS.T3 is strongly associated with protection of human individuals living in Africa and by Wang [34] and Schwenk [35] that evidenced prevalent responses of CD4 T cells against Th2R, T* and CS.T3 in individuals immunized with the RTS,S malaria vaccine.

Analysis of the CD4 T cell response to the T* sequence has demonstrated that T* contains multiple overlapping epitopes that induce CD4 T cell responses in humans and mice of diverse MHC haplotypes [30], [36], [37]. CD4 T cell clones specific for regions contained in T* have been generated from volunteers immunized with irradiated sporozoites [30]. In a clinical trial, Nardin and colleagues demonstrated antigen specificity and function of T*-specific CD4 T cell clones isolated from volunteers immunized with a fully synthetic vaccine (T1BT*)_4_-Pam_3_Cys comparable to those of clones derived from volunteers immunized with irradiated *P. falciparum* sporozoites [30], [36], [38].

T* harbors two HLA-DRβ1*04:01 (DR4) CD4 T cell epitopes as predicted originally by Parra-López [39] one highly polymorphic epitope at the N-terminus known as T*-1 that overlaps with a DR4 T cell epitope originally described by Moreno et al., [30] and one conserved epitope near C-terminus known as QNT-5 [39]. In this work, we performed a detailed characterization of the interaction of HLA-DR4 with T*-1 and QNT-5 peptides and studied the immune response to a linear peptide containing both epitopes in HLA-DR4 individuals in order to evaluate their value as potential T helper epitopes for antibody production. We found that the QNT-5 peptide forms a kinetically unstable complex with DR4. By changing the pocket 1 residue in QNT-5 from leucine to tyrosine a highly stable analogous epitope (QNT-Y) resistant to DM editing was created. QNT-Y elicited QNT-5 crossreactive T cells in DR4 transgenic mice and *in vitro* primed naïve CD4 T cells from human DR4 individuals. Although QNT-Y improved short term cellular as well as antibody responses in HLA-DR4 transgenic mice, in the long term QNT-5 was more effective in inducing these responses. These findings show that factors other than kinetic stability determine the T cell immunogenicity of QNT-5. Thus, MHC-peptide complex stability may not be a strict requirement for the generation of memory T cells that foster long-term antibody responses.

## Materials and Methods

### Human samples

Blood samples (400 mL) were taken and processed from healthy volunteers at the blood bank Hemocentro Distrital, Bogotá-Colombia (http://www.bogota.gov.co/tag/hemocentro-distrital), after the informed consent form was signed. This study was approved by Ethics committee of Universidad Nacional de Colombia Medical School – (protocol No. CE-159, 14 August 2009 Act. 12). Buffy-coats from DR4 volunteers handed to us by the blood bank were used to isolate PBMCs by density-gradient centrifugation over Histopaque (GE Healthcare). PBMCs were cryopreserved in 50% RPMI-1640 +40% FBS +10% DMSO in liquid nitrogen.

### Peptides

Synthetic peptides included in this study HA_306–318_ PKYVKQNTLKLAT; T*_326–345_ EYLNKIQNSLSTEWSPCSVT (NF-54 variant); T*-1_327–338_ YLNKIQNSLSTE; QNT-5_332–345_ QNSLSTEWSPCSVT; three QNT-5 analogues at position L335: QNT-Y (L335Y), QNT-F (L335F) and QNT-W (L335W); fourteen alanine analogues of the QNT-5 sequence (QN-Q332 to QN-T345); four QNT-5 truncated peptides (QN-334-345; QN-332-343; QN-335-343; QN-336-343); T1B a linear peptide containing the epitope T1 (DPNANPNVDPNANPNV) [31] and three copies of the B cell epitope (repeat NANP from the CS protein) [31]; the linear peptide T1BT* containing the epitope T1B sequence followed by the T* epitope (DPNANPNVDPNANPNV(NANP)_3_EYLNKIQNSLSTEWSPCSVT) and the peptide construct T1BT*-Y that contains the T1B sequence followed for the T* analogue L335Y named herein after peptide QNT-Y (DPNANPNVDPNANPNV(NANP)_3_EYLNKIQNS**Y**STEWSPCSVT) were synthesized by 21^st^ Century Biochemicals (Marlboro, MA), by the multiple-solid-phase technique, using tert-butoxycarbonyl (Boc) strategy as previously described [40]. QNT-5 alanine analogues and truncated peptides were synthesized acethylated/amide at the N- and C- terminus respectively. All peptides were purified by reverse-phase High Performance Liquid Chromatography (HPLC) on a C18 LiChrospher column (Merck, Germany). The quality of the products was assessed by analytical HPLC and mass spectrometry (MALDI-TOF). The biotin-labeled peptides HA_306–318_; T*_326–345_; T*-1_327–338_, QNT-5_332–345_ and its analogue QNT-Y, were N-terminally labeled using biotin derivative sulfo-NHS-LC-Biotin (Pierce Chemical, Rockford, Illinois). The peptide (NANP)_6_ was kindly provided by Dr. A. Moreno (Emory University).

### Soluble Recombinant DR4

Expression of soluble DR4 was performed using stable transfectants of Drosophila S2 Schenider cells, as described previously for DR1 [41]. Stable transfected cell lines were established by selection under 1.0 mg/L geneticin (Invitrogen Life technologies – California, USA) and grown in SF900 medium (Invitrogen Life technologies – California, USA) supplemented with 100 U/mL penicillin, 100 µg/mL streptomycin (Invitrogen Life technologies – California, USA), 250 µg/L amphotericin B and 2 mM L-glutamine (Invitrogen Life technologies – California, USA), at 22–24°C. Cell cultures were induced at a density of 5–10×10^6^ per mL by the addition of 0.5 mM CuSO_4_ and culture supernatant was collected 4–6 days later by centrifugation at 4000xg. Supernatant was concentrated 10-fold in a 10,000 molecular weight cut-off spiral filtration device (Millipore, Massachusetts, USA). DR4 was purified by immunoaffinity with LB3.1-conjugated protein A column, as described [42]. The final yield of DR4 was in the range of 0.2–0.5 mg/L of cultured cells.

### Recombinant HLA-DM-molecule and DR4/peptide tetramers

Recombinant HLA-DM was produced by expression in stably transfected S2-cells [43] essentially as described above for HLA-DR4 except that M2 (anti-flag tag) rather than LB3.1 was used for immunoaffinity. Biotinilated DR4/T*-1; DR/QNT-5; DR4/QNT-Y peptide complexes were produced as described by Parra-Lopez et al [39].

### DR-ELISA, direct peptide-binding assays and calculation of apparent Kd values

HLA DR4 peptide-binding studies were conducted using a modification of an ELISA-based assay [37] that consists of 3 steps: HLA-peptide complex formation, complex capture and complex detection. In this assay, HLA-peptide complex formation was achieved in 150 µL reactions containing different concentrations (0 to 4 µM) of the biotin-labeled peptides: HA_306–318_; T*_326–345_; T*-1_327–338_; QNT-5_332–345_; QNT-Y (L_335_Y) (in DMSO 10% final) and 0.05 µM purified recombinant DR4 molecules in binding buffer (100 mM citrate/phosphate buffer (pH 5.4), 0.15 mM NaCl, 4 mM EDTA, 4% NP-40, 4 mM PMSF and 40 µg/mL) for each of the following protease inhibitors: soybean trypsin inhibitor, antipain, leupeptin and chymostatin, were incubated at 37°C. After 72 hours 50 µL samples of the reactions were transferred (in duplicates) to BSA-blocked (Nunc-ELISA-Immuno-Modules Nunc-Maxisorp Loose Brand product, Denmark) previously coated with 10 µg/mL of anti-HLA-DR mAb-LB 3.1 in PBS. After 2 hours of incubation at room temperature, plates were washed with PBS, 0.05% Tween-20 and incubated for 1 hour with alkaline phosphatase-labeled streptavidin (Vector Laboratories, Burlingame, California, USA). Captured biotin-labeled peptide/DR4 complexes were revealed with 4-nitrophenylphosphate substrate (Kirkegaard and Perry Laboratories, Gaithersburg, Maryland, USA). For determining peptide binding to HLA-DR molecules, a Titertek MC Multiscan ELISA reader (Labsystems, Franklin, Massachusetts, USA) with 405 nm filter was used. By measuring the optical densities (OD_405 nm_), the amount of peptide bound was normalized with respect to the maximum observed binding.

### Peptide-binding competition assays

Peptide binding competition assays were conducted to measure the ability of unlabeled peptides (T*, T*-1, T1, QNT-5, QNT-Y, QNT-F, QNT-W; alanine peptide analogues and truncated versions of QNT-5) to compete with the biotin-labeled indicator peptide HA_306–318_ PKYVKQNTLKLAT for binding to DR4 molecules. Critical residues of QNT-5 for binding to DR4 were defined by the use of a series of single substitution alanine QNT-5 analogues. For the competition assay unlabeled peptides at concentrations ranging from 0 to 100 µM were mixed in binding buffer together with biotin-labeled HA peptide (0.025 µM) and DR4 molecules (0.05 µM) in a final 150 µL volume and the reactions incubated for 72 hours at 37°C. Competition assays were revealed using DR-ELISA described above, IC_50_ was calculated by minimal squares and percentages of DR4 binding inhibition were calculated by using the formula: 100×[1- (ΔOD_405 nm_ in the presence of competitor)/OD_405 nm_ in the absence of competitor peptide].

### Assay of dissociation kinetics

Association reactions of DR4 with each of the biotin-labeled peptides T*-1; QNT-5; QNT-Y and HA were performed by incubation of 1 µM DR4 molecules with 10 µM biotin labeled peptide in 400 µL binding buffer for 72 hours at 37°C. Biotin-labeled peptides were removed from reactions by gel filtration using Sephadex G50 columns. The reaction was then diluted 1∶2 with binding buffer and split in two tubes (400 µL each). The dissociation reactions in presence and absence of HLA-DM were started by adding to one tube dissociation mix having 10 µM unlabeled HA peptide and 1 µM HLA-DM and to the second tube only 10 µM unlabeled HA peptide in a final volume of 2 mL. At different time points, 200 µL aliquots of each dissociation reaction (+/− HLA-DM) were transferred into tubes having 20 µL of 1 M Tris pH 8.0 that were frozen immediately in ethanol/dry ice and stored at −70°C until use. The thawed dissociation mixtures were incubated in anti-DR1 Ab LB3.1 precoated 96-well Lumitrac 600 white plates (USA Scientific, Ocala, FL, USA) at 4°C for 3 h, washed three times with PBS +0.05% Tween-20, incubated with Europium-streptavidin at 37°C for 1 hour, washed again, and then mixed with Europium enhancement solution to release EU3+ (PerkinElmer, Shelton, CT, USA). A Victor plate reader (PerkinElmer, Shelton, CT, USA) was used to read the time-resolved fluorescence of EU3+. The dissociation curve was fitted to single-phase exponential decay with constraint 100% bound at time 0 in GraphPad Prism 5 (GraphPad software, San Diego, CA, USA) to determine the off rate, k_off_, and half-life.

### Molecular modeling

The X-ray crystal structures of a complex of a human alpha/beta-TCR influenza HA antigen peptide and DR4 (PDB code 1J8H) [44] and DR4 with a peptide mimetic inhibitor of antigen presentation by HLA-DR class II MHC molecules (PDB code 1D5Z) [45] were used as template for further modeling. The total energy interaction of HA or peptide mimetic inhibitor with DR4 were determined first without including any further refinements using docking software (Accelrys Software Inc., San Diego, California, USA) run on an Indigo 2 Station (Silicon Graphics, Sunnyvale, California, USA). In order to obtain the energetic interaction of minimized peptide structures interacting with DR4, the Discovery 3 program (Accelrys, San Diego, California, USA) was used for simple minimization strategy using 20000 steps and 0.0001 Å RMSD. For modeling peptides QNT-Y and QNT-5 interacting with DR4, amino acids side chains of template peptides were replaced by QNT-5 (LSTEWSPCS) and QNT-Y (YSTEWSPCS) side chains and the free energy of interaction of side chains of amino acid residues of both peptides with corresponding DR4 pockets (reported as explicit van der Waals (VDW) energy values) were determined with and without further refinements. To analyze putative anchor residues at P1 in modeled peptides QNT-5 and QNT-Y, the intermolecular energy of residues occupying P1 on template peptides HA and peptide mimetic inhibitor (Y and cyclohexylalanine (Cha) respectively) was compared to those of L and Y acting as P-1 anchoring residues of the QNT-5 peptide sequence.

### Immunization of DR4 transgenic mice

HLA-DR4 transgenic mice from Taconic Farms housed at the University of Massachusetts in Worcester MA animal facility were used in the experiments. Animals were handled and euthanized according to University of Massachusetts Medical School Institutional Animal Care and Use Committee approved through the Animal Protocol A-1419. Three groups of four DR4 transgenic mice (HLA-DRα1*01:01/DRβ1*04:01) were immunized subcutaneously at the base of the tail with 50 µL of an emulsion containing 50 µg of peptide T1B, T1BT* or T1BT*-Y in Montanide ISA 720 as adjuvant or adjuvant alone as control. Mice were immunized with two or three doses of the peptides or adjuvant (20 days apart). One hundred to two hundred microliters of blood was obtained from the facial vein of each experimental mouse by a trained technician before each immunization.

### Antibody titer determination

Anti-*P. falciparum* CS repeat immunoglobulin G (IgG) titers were determined in individual serum samples by enzyme-linked immunosorbent assay (ELISA) using immobilized (NANP)_6_ peptide, peroxidase-labeled species-specific anti-IgG antibody, and 2,2-azinobis(3-ethylbenzthiazolinesulfonic acid) (ABTS) substrate. Antibody titers were defined as the highest serum dilution that yielded an ABTS optical density greater than the optical density observed for the mean plus three standard deviations of pre-immune serum. IgG subtypes were determined by ELISA using biotin-labeled monoclonal antibodies to IgG1, IgG2a, IgG2b and IgG3 (Biolengend) followed by peroxidase-labeled streptavidin and ABTS. Each assay plate included positive control wells covered with mouse IgG, IgG1, IgG2a, IgG3b or IgG3 (all from BD Biosciences). A control monoclonal antibody (2A10) was used also as a positive control for antibody responses to the *P. falciparum* CS repeats [46].

### Determination of IFN-γ responses by ELISPOT in splenocytes of DR4 transgenic mice immunized with T1BT* or T1BT*-Y

IFN-γ ELISPOT was performed using IFN-γ ELISPOT Kit as manufacturer instructions (eBiosciences, San Diego, CA, USA) in PVDF plates (Millipore, Billerica, MA, USA). In brief, plates were covered with 100 µL of capture antibody and after overnight incubation at 4°C washed and blocked with RPMI-1640 +10% FCS. Subsequently, 10^6^ splenocytes in 100 µL of RPMI supplemented with 10% FCS were seeded per well and peptides (T*-1, QNT-5, QNT-Y, HA) at a final concentration of 10 µg/mL added. After 48 hours incubation at 37°C, 5% CO_2_ plates were washed 3 times with 1× PBS, 0.05% Tween-20 and cytokine revealed by successive incubation with detection antibody, wash, peroxidase-HRP incubation, wash and final visualization with AEC substrate. Plates were scanned and spots counted with the help of an immunospot Analyzer (CTL-immunospot S6, Cellular Technology Limited, USA).

### Quantification of human CD4 T cells specific for T*-1; QNT-5 and QNT-Y epitopes generated from naïve CD4 T cells primed in vitro with T1BT* and T1BT*-Y

Specific cell lines against T1BT* and T1BT*-Y peptide constructs were generated from naïve CD4 T cells from a HLA-DRβ1*04:01:01 donor using as APCs autologous monocyte derived dendritic cells (DCs) as described by Moser and colleagues [47]. CD4 T cells were purified using a naïve CD4 T cell isolation kit as manufacturer instructions (Miltenyi Biotec, Germany). DCs were pulsed with 3.75 mM of peptides T1BT* or T1BT*-Y. DCs were matured with TNF-α (25 ng/mL). Cells were co-cultured at 37°C, 5% CO_2_ for 14 days and re-stimulated at 1∶10 ratio with mature dendritic cells pulsed with 3.75 mM of the same peptide used to prime. Seven days after the cells were stained with fluorescent DR4/T*-1; DR4/QNT-5 and DR4/QNT-Y tetramers assembled by stepwise addition of streptavidin-PE (Caltag, Life technologies – California, USA) to biotinylated HLA-DR4–peptide complexes at a final molar ratio of 1∶4 as described [41]. T cells were incubated with tetramers for 4 hours at 37°C followed by 20 min incubation with cell surface markers: anti-CD3 PE-Cy7, anti-CD4 PE-Texas red (Beckman Coulter, Palo Alto CA. USA), anti-CD45RO FITC, anti-CD62-L PECy5 (all from BD Biosciencies. San Diego CA. USA) on ice before washing with ice-cold buffer. Tetramer and Ab binding were determined using an FACS Aria II flow cytometer (BD Biosciences).

### Statistical analysis

Raw ELISPOT data was analyzed by Kruskal-Wallis test with Dunn's Multiple Comparison Test to compare numbers of IFN-γ spot forming units (IFN-γ SFU) between splenocytes of mice immunized with peptide and control mice (vaccinated with adjuvant only) upon stimulation in vitro with various assay antigens (T1BT*, T1BT*-Y, T*-1; QNT-5, QNT-Y, T1 and HA_306–318_). Anti-repeat antibody titers were analyzed with Mann Whitney test.

## Results

### Identifying anchoring residues for QNT-5 binding to DR4

**Figure 1:**
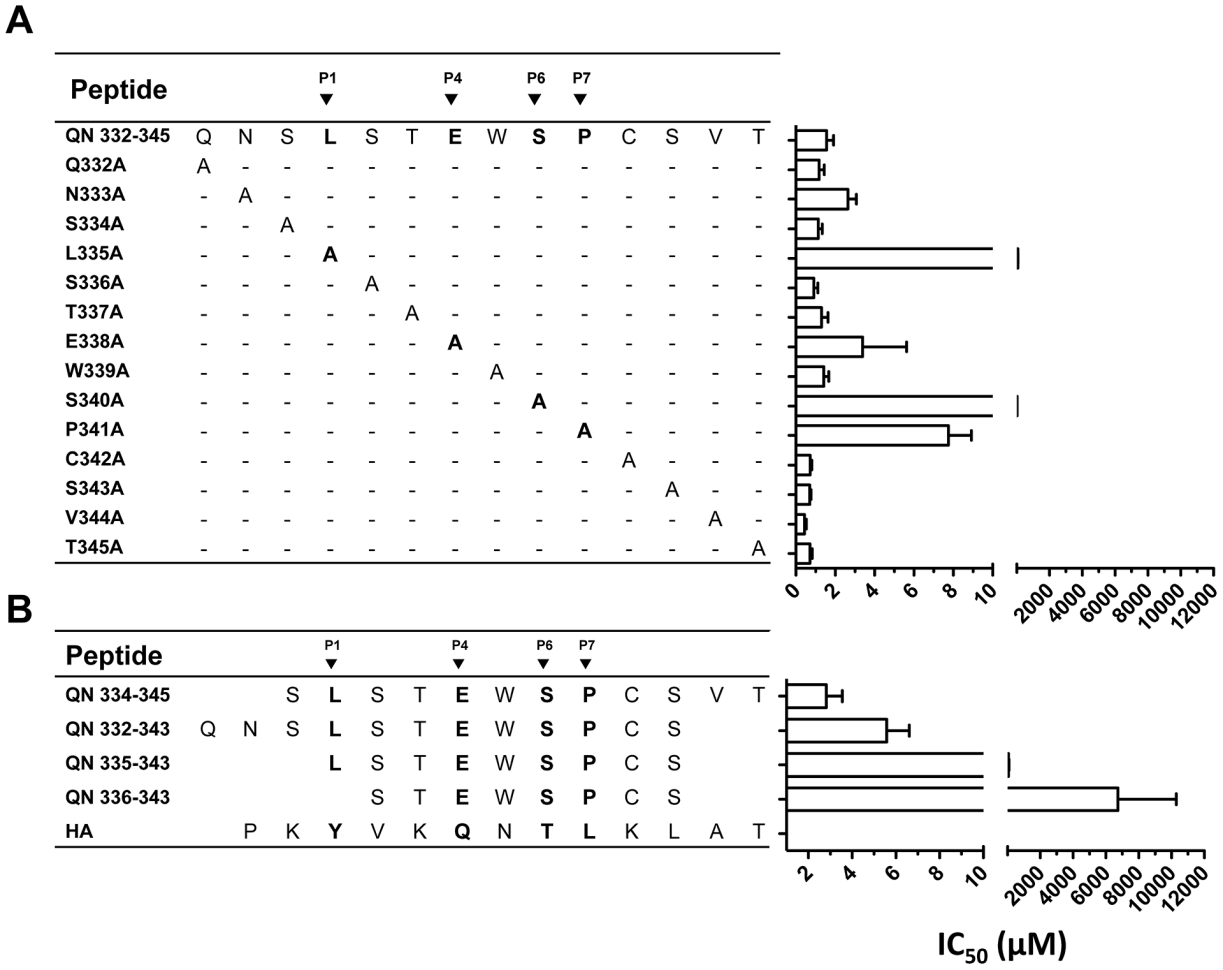
Identification of residues in QNT-5 critical for binding to DR4. A series of QNT-5 analogues with single alanine substitutions (A) and N- and C-terminal truncated QNT-5 peptides (B) were synthesized and tested at concentrations ranging from 0 to 100 µM for their ability to bind to DR4. Relative binding was measured in a binding inhibition assay. The concentration of each alanine analogue or truncated peptide required to reduce the binding of a biotin-labeled test peptide to 50% (IC_50_) is shown. The test peptide was a biotinylated version of HA (_306_PKYVKQNTLKLAT_318_), a peptide from influenza hemagglutinin that binds strongly to DR4. Under the conditions used in this set of experiments, the IC_50_ for the HA peptide was 0.31±0.05 µM (Table S1). Representative findings from 3 experiments performed are shown (each point was performed in duplicate). Bars represent SD.

In a previous study we described two overlapping HLA-DR4-binding epitopes within the universal CD4 T cell epitope T* from *P. falciparum* [39]. MHC and T cell interactions were described for T*-1, an epitope near the N-terminus of the T* peptide (CS_327–338_, YLNKIQNSLSTE). A second epitope termed T*-5 (_334_SLSTEWSPCSV_344_) was mapped near the C-terminus of the T* peptide, but was not fully characterized. Previous publications that analyzed antigen-specific CD4 T cell clones isolated from DR4 individuals vaccinated with a synthetic peptide vaccine (T1BT*)_4_-Pam_3_Cys found that some of these clones recognized the core SLSTEWSP sequence contained in T*-5 [36], [38]. To perform a fine mapping of the C-terminal epitope, and to analyze side-by-side its HLA-DR4-binding interactions with those of T*, T*-1, and control tight-binding viral peptide HA derived from influenza heamaglutinin, we synthesized a series of peptide variants based on QNT-5 (_332_QN**SLSTEWSPCSV**T_345_) (Table 1). The QNT-5 peptide corresponds to the T*-5 sequence elongated by two and one residues at the N- and C- terminus of the core respectively. We performed binding competition assays between biotin-labeled HA and a set of unlabeled single alanine substitution analogues and truncation variants of QNT-5. IC_50_ values for these peptides are shown in Figure 1A and Table S1. Alanine substitution of L_335_ or S_340_ greatly decreased binding of QNT-5 (as indicated by high IC_50_ values) with smaller effects observed upon substitution of E_338_ and P_341_. These effects are consistent with a binding frame in which L_335_, E_338_, S_340_ and P_341_ of the QNT-5 peptide (black arrowheads in Figure 1A) bind in the canonical P1, P4, P6, and P7 pockets of HLA-DR4 [20], [48], [49]. As previously observed for other [20], [48]–[50] HLA-DR4-binding peptides alanine substitution at the P9 position (S_343_) did not have a significant effect on QNT-5 binding (Figure 1A). Truncation analysis (Figure 1B) suggests that peptides need to extend at least to the P(-1) position, since deletion of S_334_ (QN335-343) significantly reduced binding to HLA-DR4, even though the S_334_A substitution did not have a significant effect (Figure 1A).

**Table 1:** Aminoacid sequence of T* and DR4 epitopes used in this study.

Peptide	Aminoacid number	Aminoacid sequence																			
T*	326–345	E	Y	L	N	K	I	Q	N	S	L	S	T	E	W	S	P	C	S	V	T
T*- 1	327–338		Y	L	N	K	I	Q	N	S	L	S	T	E							
QNT-5	332–345							Q	N	S	L	S	T	E	W	S	P	C	S	V	T
QNT-Y	332–345							Q	N	S	Y	S	T	E	W	S	P	C	S	V	T
HA	306–318	P	K	Y	V	K	Q	N	T	L	K	L	A	T							

### Apparent binding affinity (K_d_) of peptide complexes in presence and absence of HLA-DM

Previously we reported that T*-5 peptide bound to HLA-DR4 more weakly than the T*-1 peptide as evaluated by a competition binding assay, with IC_50_ values of 0.9 and 0.2 µM, respectively [39]. To evaluate the binding affinity more precisely and to compare T*-1 and QNT-5 directly, we performed direct binding assays using biotinylated variants of T*-1 and QNT-5 with a fixed concentration of HLA-DR4 (see Methods). T*-1 bound more tightly to HLA-DR4 than did QNT-5, with apparent *K_d_* values of tenfold lower, ∼42 nM versus ∼504 nM, respectively (Table 2 and Figure S1). Apparent *K_d_* was similar in presence of HLA-DM (∼51 and ∼714 nM respectively), as expected since HLA-DM acts as a peptide-exchange catalyst but does not alter the binding equilibrium.

**Table 2:** Interaction with HLA-DRβ1*04:01 of HA and DR4 epitopes of T*.

Peptide	*K_d_* (nM) DM-	*K_d_* (nM) DM+	Half-life (min)	Half-life DM (min)	DM-sus (10^−3^ min^−1^ µM^−1^) *(c)*
T*	248±75.1 *(a)*	123.15±1.05	ND	ND	ND
T*-1	42.61±22.84	51.59±9.30	3831 *(b)*	294	2.14
**QNT-5**	504.23±86.23	714.1±3.90	304.8	105	4.33
**HA**	42.81±39.93	44.77±39.77	37200	5472.6	0.11
**QNT-Y**	25.47±10.70	35.67±20.50	8307	2589	0.18

a) Apparent equilibrium binding affinity.

b) Dissociation half-life from single exponential decay.

C)DM susceptibility. DM-sus was calculated by (k_off,DM_ - k_off,in_)/[DM] as described by Yin et al., [4] where k_off,in_ = ln2/_Half-life_, k_off,DM_ = ln2/_Half-life DM_, and [DM]  = 1 µM.

ND. Not done.

### DR4/QNT-5 peptide complexes are highly unstable

**Figure 2:**
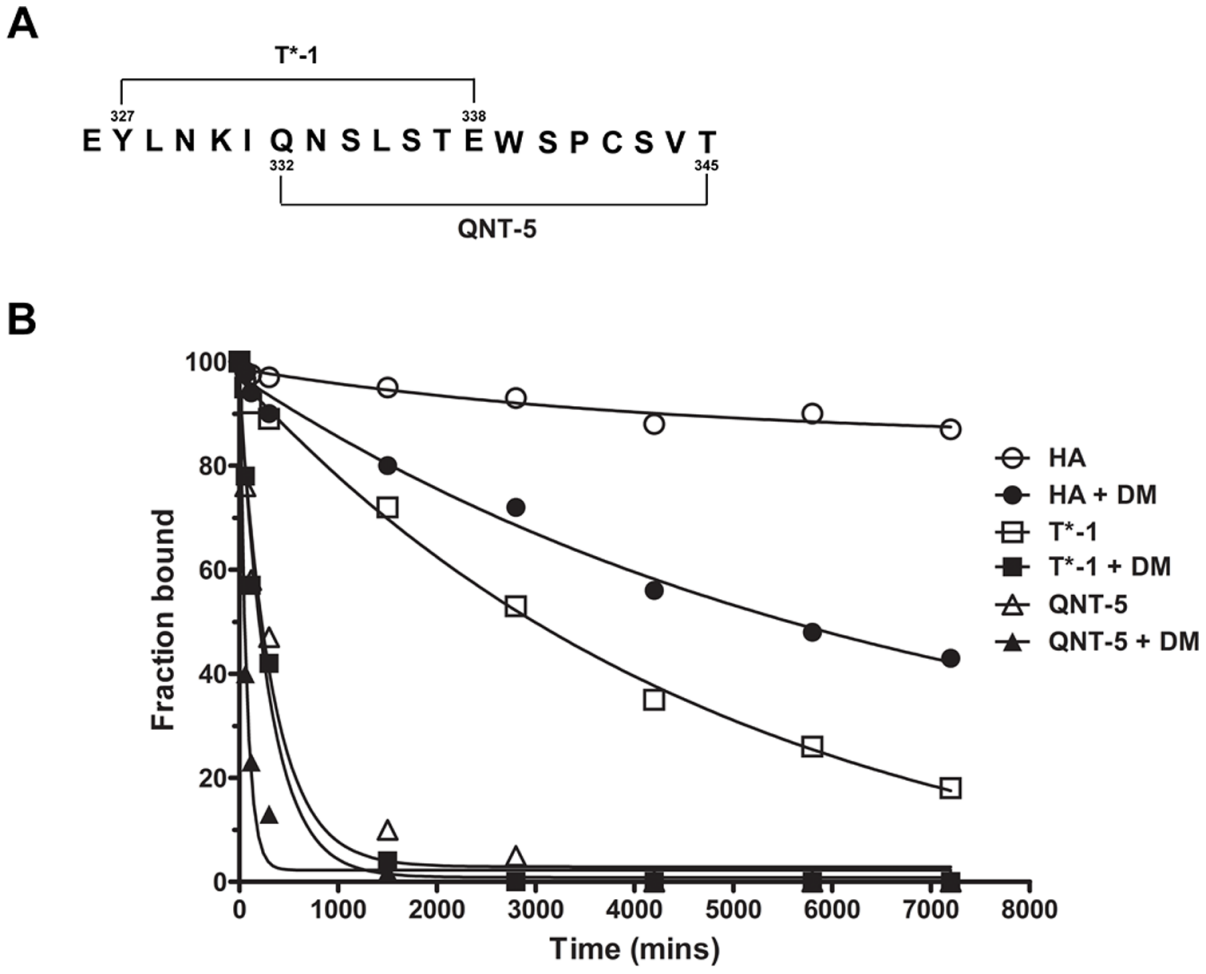
Dissociation kinetics from DR4 of HA, T*-1 and QNT-5 peptides measured in presence and absence of HLA-DM. (A) Amino-acid sequence and location of T*-1 and QNT-5 epitopes in T*. (B) Characterization of the dissociation behavior of peptide MHC complexes formed after 72 h of complex formation. The curves shown represent single or double exponential decays that fit the data. Filled symbols represent the decays values of the 3 DR4-peptide complexes in the presence of HLA-DM. Empty symbols represents the decay values of the complexes in the absence of HLA-DM. A representative experiment from 2 experiments performed is shown (each time point was carried out in duplicate).

We also evaluated the kinetic stability of purified DR4/T*-1 and DR4/QNT-5 complexes (Figure 2, Table 2). DR4/T*-1 formed a stable complex with half-life ∼3800 min at 37°C, although dissociation was still much quicker than for the prototypical tight-binding viral peptide HA (half-life 37,000 min). DR4/QNT-5 formed a much less stable complex, with half-life ∼300 min. HLA-DM increased the dissociation of both DR4/T*-1 and DR4/QNT-5 (half lives of ∼300 and ∼100 min respectively).

### DR4/QNT-5 peptide complex instability can be reversed by a tyrosine in QNT-5 occupying P1

**Figure 3:**
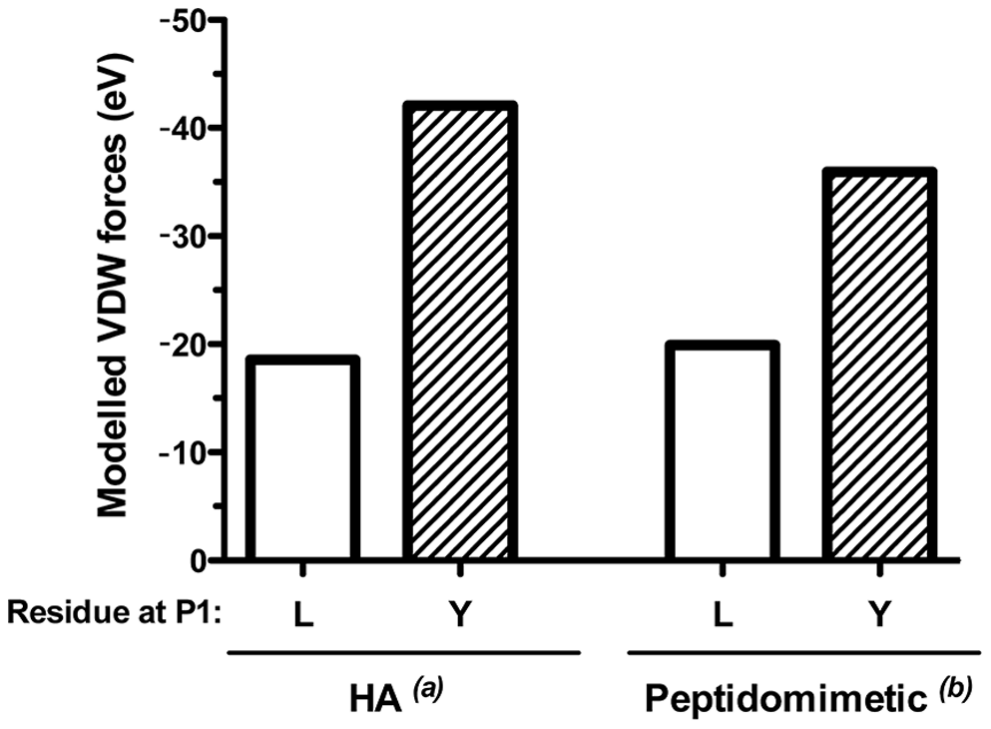
VDW energy value for the hydrophobic interactions at P1 between DR4 and QNT-5 or an analogue of QNT-5 with a L_335_Y substitution at P1 (peptide QNT-Y). VDW value after the computational docking of peptides QNT-5 and QNT-Y into an MHC (DRβ1*04:01:01) structure, originally determined for the HA peptide and peptidomimetic inhibitor complexes, with a tyrosine*^(a)^* [44] (left panel) and a cyclohexylalanine*^(b)^* side chain at P1 [45] (right panel), respectively. The height of the white- and dashed-boxes represents the VDW free energy value expressed in eV for QNT-5 (L) with the anchor residue L_335_ at P1 and for QNT-Y (Y) with Y_318_ at P1, respectively.

**Figure 4:**
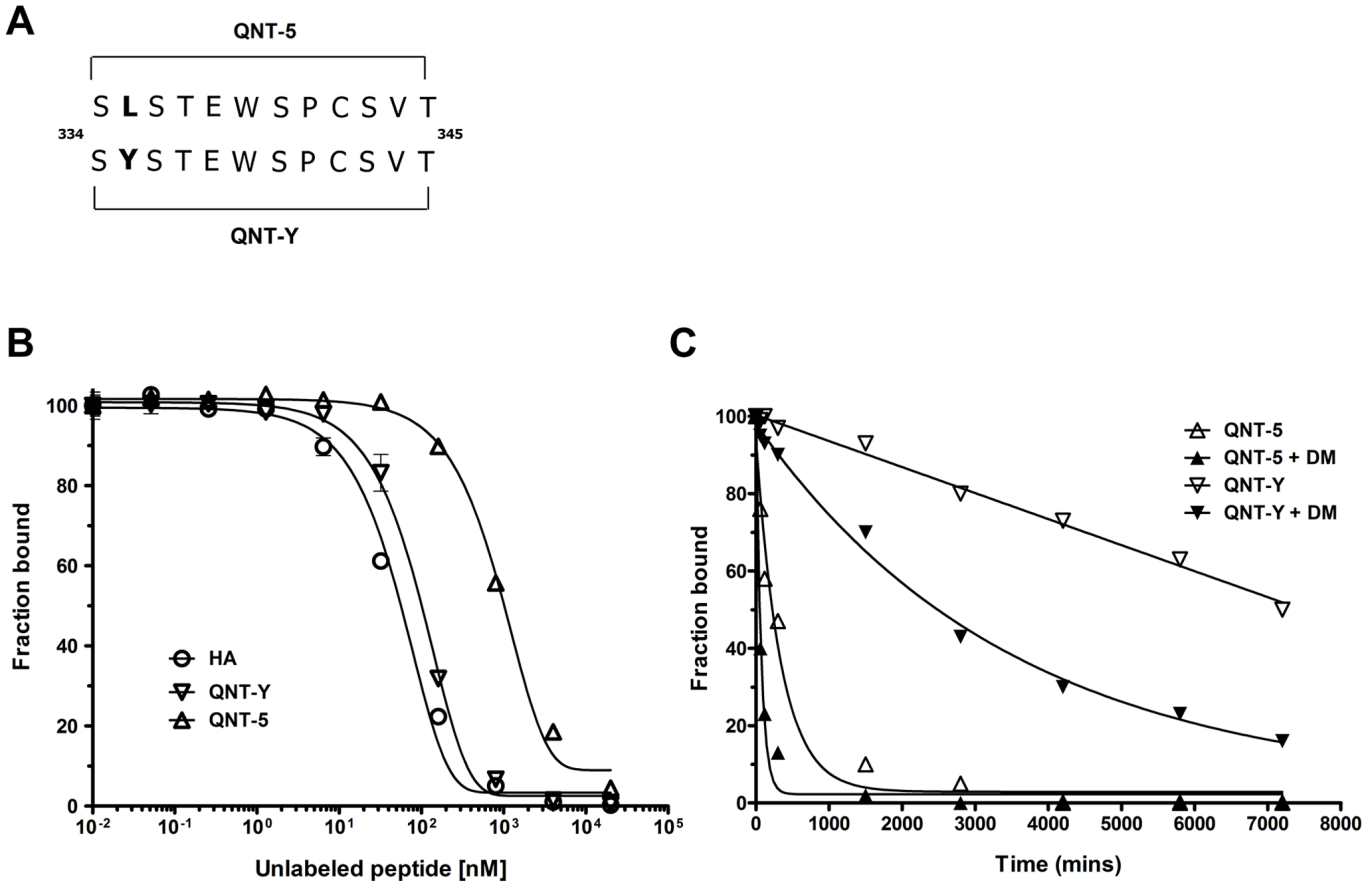
Binding activity of QNT-Y and stability of the DR4/QNT-Y peptide complex. (A) Amino-acid sequence of QNT-5 and QNT-Y peptide analogue. (B) Competition binding assay for QNT-5, QNT-Y and HA. The plots show the binding inhibition of the biotinylated HA_306–318_ peptide to DR4 using increasing amounts (0 to 20 µM) of unlabeled peptides to calculate the concentration of each peptide required to reduce the binding of a biotin-labeled test peptide to 50% (IC_50_). Under the experimental conditions used here, the IC_50_ for the HA peptide was ∼172 µM. Representative results from 1 of 3 experiments performed are shown (each point was performed in duplicate). (C) Dissociation kinetics of the QNT-5 and QNT-Y peptides from DR4, measured in the presence (filled symbols) or absence (empty) of HLA-DM. A representative experiment of 3 performed is shown (each time point was performed in duplicate). Bars represent SEM.

Published results from Sant and others suggest that immuno-dominance in CD4 T cell responses is primarily an intrinsic property of peptide-class II complex stability that can be modulated by manipulating MHC-peptide interactions [22]. The unstable behavior DR4/QNT-5 led us to predict that it would be poorly immunogenic and that this could be solved by the rational modification of MHC-peptide complex stability. The T*-1 sequence is highly variable, with more than 28 variants reported [51]–[55]; in contrast, QNT-5 (QNSLSTEWSPCSVT) is highly conserved throughout several *Plasmodium* species and harbors a sequence that overlaps with the region II sequence of the CS protein (underlined) that is crucial for the entrance of sporozoites into hepatocytes [56], [57]. These attributes of QNT-5 and the fast off rate of DR4/QNT-5 complexes even in absence of HLA-DM (see Table 2) prompted us to focus our search on the structural features of this sequence responsible for its unstable behavior using a combination of computational and experimental approaches. First, we analyzed the QNT-5 sequence using Propred [58], which is a matrix-based algorithm that can be used for predicting peptide binding to many MHC class II alleles, including the HLA-DRβ1*04:01 allele. Because this algorithm typically identifies strong allele-specific preferences at positions P1, P4, P6 and P9, based on docking data, we oriented the analysis to search for amino acid substitutions at P1 that would improve binding of QNT-5 to DR4. The results of these analyses predicted that the QNT-5 peptide sequences with aromatic residues Y, F and W substituted for L_335_ at P1 would enhance peptide binding (data not shown). Second, a docking program for evaluating the probability of MHC-peptide complex formation was used to determine the explicit VDW energy value of the hydrophobic interactions at each putative residue of the QNT-5 and QNT-Y (a QNT-5 analogue having L_335_Y substitution at P1) peptide sequences occupying the P1, P4, P6 and P9 pockets at the peptide-binding groove of DR4 (β1*04:01). After computational docking into a MHC structure originally determined for a HA peptide complex [44], significant differences between the QNT-5 and QNT-Y peptides were observed only at the P1 pocket, as expected. The free energy of this residue at P1, estimated by the VDW force value for QNT-5 with the anchor residue L_335_ at P1, was −18.56 eV, which is substantially higher than that of Y_318_ with −42.06 eV (Figure 3 and data not shown). The same analyses were performed using a different MHC structural model consisting of a complex with a peptidomimetic inhibitor that contains a cyclohexylalanine side chain at P1 [45]. The same pattern was observed, with free energies of −19.91 eV after minimization for L_335_ at P1 and −35.96 eV for Y_318_ (Figure 3 and data not shown). Thus, Y_318_ was predicted to be more favorable in the P1 pocket than L_335_, regardless of the starting model used for the calculations. This information, together with the results of the algorithm prediction of a peptide analogue of QNT-5 containing the L_335_Y substitution at P1 (QNS**Y**STEWSPCSVT), hereafter named QNT-Y (Figure 4A), which predicted a higher stability of Y over L_335_ at P1 in DR4, led us to hypothesize that the DR4/QNT-Y peptide complex might exhibit greater stability than the DR4/QNT-5 complex.

In order to evaluate experimentally whether a P1 substitution could stabilize QNT-5 peptide, we performed competition binding assays (IC_50_) for QNT-5, QNT-Y and the control tight-binding HA peptide (Figure 4B). QNT-Y binds to DR4 with a relative affinity ∼10-20-fold higher than QNT-5 (IC_50s_ ∼130 nM vs. ∼1700 nM) (range values 124.2–139.9 and 1528–1848 (95% C.I.) respectively). The higher affinity of QNT-Y for DR4 was evaluated also in a direct binding assay using biotinylated peptides, in the presence and absence of HLA-DM. Compared to QNT-5, the binding affinity of QNT-Y was increased ∼20-fold with apparent *K_d_* values with and without HLA-DM of ∼36 and ∼25 nM, respectively (Table 2). Finally, having found that QNT-Y exhibited a higher affinity for DR4, we sought to examine the impact of the L_335_Y change at P1 on the kinetic stability of the DR4/QNT-Y complex as well as the sensitivity of this peptide complex to HLA-DM editing. We observed a ∼25-fold increase in the half-life of DR4/QNT-Y compared to DR4/QNT-5 complexes in presence or absence of HLA-DM (lifetimes of ∼2,500 and ∼8,300 min, respectively (Figure 4C and Table 2)). These results clearly demonstrate that the L_335_Y substitution at P1 reverses the unstable behavior of the DR4/QNT-5 peptide.

### In DR4 transgenic mice a linear peptide malaria vaccine candidate containing QNT-Y elicits higher anti-CS repeat antibody titers than a peptide containing QNT-5 but the effect is transient and long-term responses are reduced

**Figure 5:**
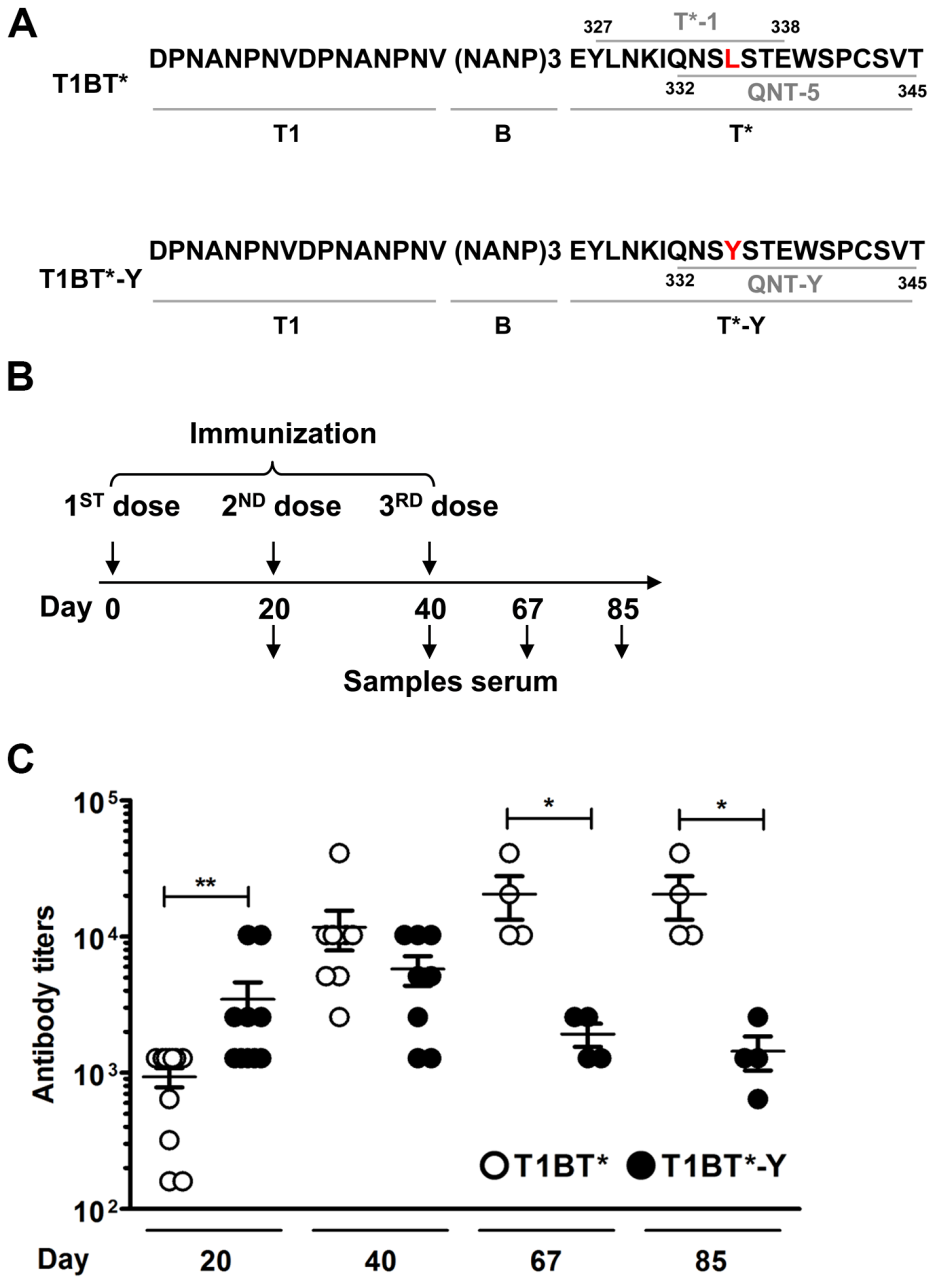
Long term quantitation of anti-(NANP)_6_ Ab responses by ELISA in mice vaccinated with T1BT* or T1BT*-Y peptides. (A) Amino-acid sequences of T1BT* and T1BT*-Y polypeptides used for vaccination of HLA-DR4 transgenic mice. T1BT* comprise T1 (a T cell epitope from the 5′minor repeat region of *P. falciparum* CS protein [31]), B (three copies of immune-dominant B-cell repeat epitope (NANP) from *P. falciparum* CS protein [97]) and the NF54 variant of T* epitope that includes T*-1_327–338_ YLNKIQNSLSTE and QNT-5_332–345_ QNSLSTEWSPCSVT with L_335_ highlighted in red. T1BT*-Y is identical to T1BT* except that QNT-5_332–345_ QNSYSTEWSPCSVT harbors the single amino-acid substitution L_335_Y (in red). (B) Immunization scheme indicating the days when serum samples were collected. (C) Anti-(NANP)_6_ antibody titers in serum samples of mice immunized with T1BT* (open) or T1BT*-Y (black circles) during the course of the immunization protocol. The mean anti-(NANP)_6_ Ab titers correspond to the average titer determined in the sera of groups of 3 DR4 transgenic mice immunized with T1BT* or T1BT*-Y in 3 independent experiments. (*) p<0.05; (**) p<0.001 Mann Whitney test, mean with SEM (standard errors of the mean) bars are shown.

The identification of QNT-5 variants with different kinetic stabilities provides a useful system to analyze the role of epitope stability in CD4 T cell immunodominance hierarchy and the capacity to function as T helper epitopes in the anti-(NANP)_3_ antibody response. To determine the relative T helper activity of QNT-5 and QNT-Y two peptides were synthesized, T1BT* and T1BT*-Y (having the stable L_335_Y substitution in QNT-5 (Figure 5A)). Both peptides have in common the T1B sequence that contains the minor T1 epitope [19], [31] and three copies of the NANP repeat that is the major antibody epitope in the *P. falciparum* CS protein. Although T1 functions as a T cell epitope in DR4 haplotype malaria-immune individuals [19], [31] we do not expect significant T cell responses to T1 in DR4 transgenic mice immunized with T1BT for two reasons: *(i)* in previous studies, a DR4 individual immunized with a construct containing T1BT* developed T cell response to T* but not to T1 [36], [38] and *(ii)* contrary to QNT-5 and QNT-Y (IC_50_ ∼25 and ∼500 respectively, Table 2), T1 binds very weakly to DRβ1*04:01 (IC_50_ ∼18000 nM, not shown). The “B” sequence functions as a reporter for anti-CS repeat antibody responses, although “T1” sequences are also recognized. We used this construct because previous studies demonstrated that T1BT* is highly immunogenic in humans [36], [38] and also because T1BT*-immunized mice are protected when challenge with sporozoites in a murine malaria model for *P. falciparum* [59].

DR4 transgenic mice were immunized with T1BT* and T1BT*-Y following a typical vaccination protocol, with three immunization doses spaced 20 days apart collecting blood samples before each immunization (days 0, 20, 40) and at days 67 and 85 after first dose (Figure 5B). Antibody titers throughout the immunization scheme shown in figure 5B are summarized in Figure 5C and Table 3. Figure 5C shows that 20 days after the first dose, anti-(NANP)_6_ antibody titers in sera of mice immunized with peptide T1BT*-Y (filled circles) were significantly higher (p<0.001) than those detected in mice immunized with T1BT* (open circles). However, anti-(NANP)_6_ antibody titers in sera of both groups of animals were similar by the second dose, and antibody titers in mice vaccinated with T1BT*-Y did not sustain in the long term. Two months after the third dose the anti-(NANP)_6_ antibody titers in mice vaccinated with T1BT*-Y were significantly lower (p<0.05) than in mice vaccinated with T1BT* (Figure 5C). The role of T* as a T helper epitope in the antibody response to (NANP)_6_ elicited by immunization with T1BT* or T1BT*-Y was corroborated by a lack of antibody responses in DR4 transgenic mice immunized with T1B (Table 3). Using the same immunization scheme shown in figure 5B, only one of four DR4 transgenic mice immunized with a linear T1B peptide developed anti-(NANP)_6_ antibody response, and this response was very weak with titer 1∶80 as compared to titers over 1∶1000 in T1BT* and T1BT*-Y immunized mice (Table 3). This led us to conclude that anti-(NANP)_6_ antibody responses observed upon immunization of DR4 transgenic mice with T1BT* or T1BT*-Y are mostly associated with help by T*.

**Table 3:** Anti-(NANP)_6_ antibody responses in DR4 transgenic mice after three subcutaneous immunizations with synthetic peptides in Montanide ISA 720.

Immunogen	Responders/Total	GMT*	Ab Titer Range^#^
T1BT*	4/4	17222	10240–40960
T1BT*-Y	4/4	1810	1280–2560
T1B	1/4	80	<1∶80–80

*Geometric Mean titers (GMT) and ^#^Range of antibody titers in the group of mice immunized with peptides indicated in the first column.

### Isotype specificity of the anti-repeat response elicited by vaccination with T1BT* and T1BT*-Y

**Figure 6:**
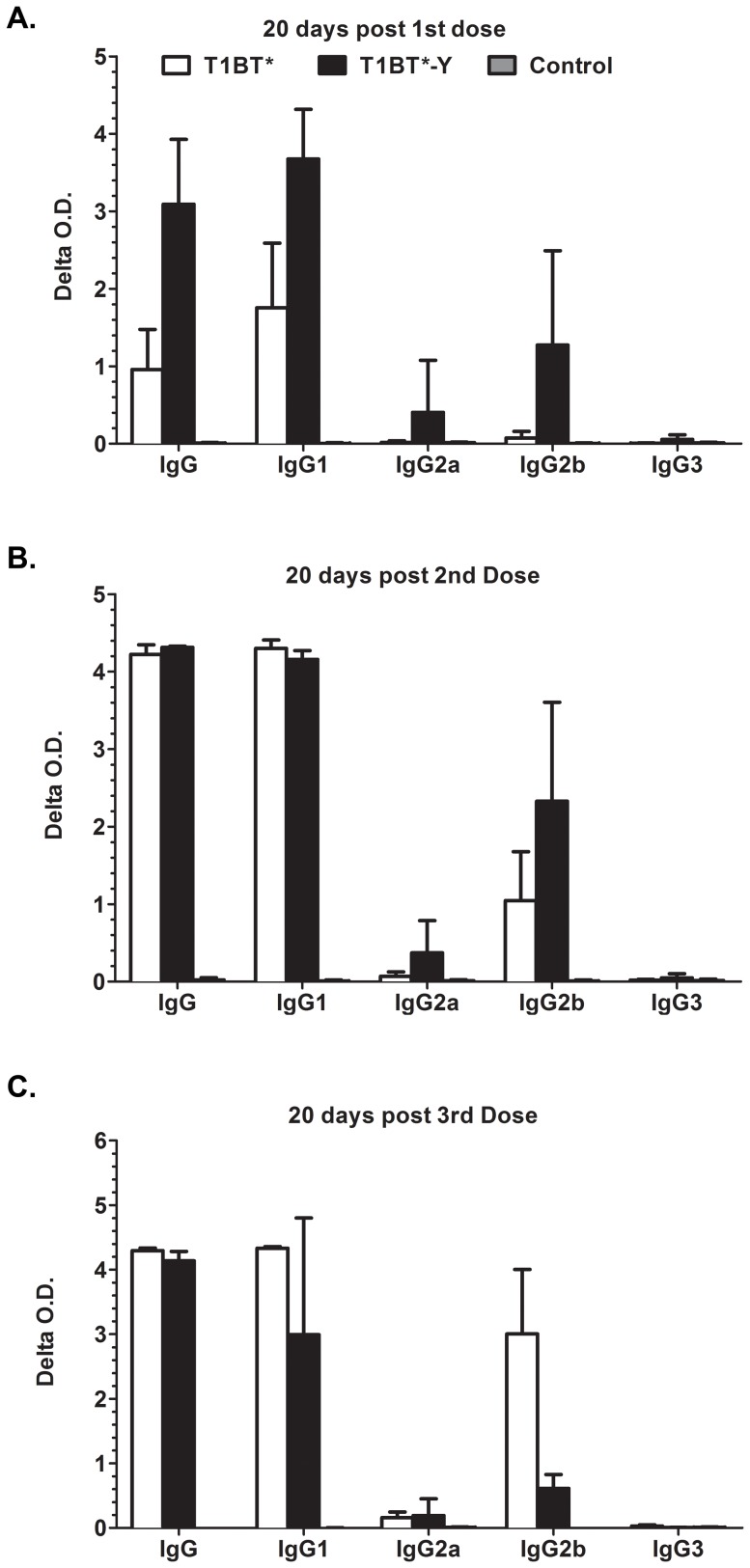
IgG Isotype responses in T1BT* and T1BT*-Y immunized mice. IgG subtype of anti-(NANP)_6_ antibody responses elicited in DR4 transgenic mice twenty days after the first (A), second (B) and third dose (C) of T1BT* (white bars); T1BT*-Y (black bars) peptides or Montanide ISA 720 (grey bars). The bars indicate mean delta O.D. (optical density serum in wells coated with (NANP)_6_ minus PBS wells) obtained with DR4 transgenic serum (1∶80 dilution) incubated with (NANP)_6_ peptide-coated ELISA plates and reacted with IgG subtype-specific antibodies. Serum samples were tested individually and means and standard deviation for the group are shown.

IgG isotype responses to T1BT* and T1BT*-Y in immunized mice are presented in Figure 6. Mice immunized with these constructs produce an early IgG1 response, with T1BT*-Y inducing a stronger response. T1BT*-Y-immunized mice also produced IgG2b and a low levels of IgG2a. Following a second dose total IgG and IgG1 responses have similar strength in both groups of mice, and IgG2b responses are now also observed in T1BT* immunized mice (Figure 6B). Finally, after a third dose the IgG2b antibody responses in T1BT*-Y are appreciable lower than in T1BT* immunized mice (Figure 6C). Overall this pattern is similar to that observed for the IgG titers reported in Figure 5C. None of the constructs induced IgG3 responses to (NANP)_6_ peptide.

### Peptides containing QNT-5 and QNT-Y epitopes elicit IFN-γ responses in DR4 transgenic mice

**Figure 7:**
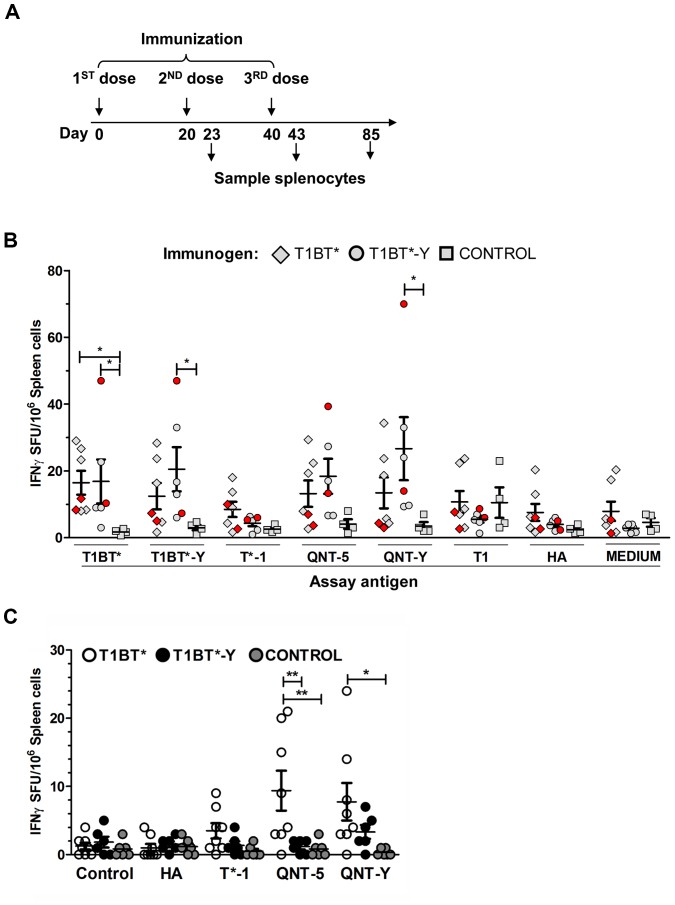
Quantitation of IFN-γ secreting cells in the spleens of mice after vaccination with T1BT* or T1BT*-Y by ELISPOT. (A) Immunization scheme indicating the days when splenocytes for ELISPOT were collected. (B) The graph shows the mean number of splenocytes producing IFN-γ per 1×10^6^ cells from mice immunized with T1BT* (diamonds), T1BT*-Y (filled circles) or adjuvant/PBS (squares) after stimulation for 48 h *in vitro* with the assay antigens (T1BT*, T1BT*-Y, T*-1, QNT-5, QNT-Y, T1 and HA (10 µg/mL each)). The *p* values are relative to control mice immunized with PBS/adjuvant; * p<0.05. Kruskal-Wallis test with Dunn's Multiple Comparison Test. The IFN-γ SFU at day 20 from mice immunized with only 2 antigen doses is shown in red. Mean with SEM (standard error of the mean) bars are shown. (C) IFN-γ secreting cells quantified by ELISPOT at day 85 in spleens of DR4 transgenic mice immunized with T1BT* (open circles), T1BT*-Y (black) or adjuvant/PBS (gray) after stimulation for 48 h in vitro with media (control) or the assay antigens HA; T*-1, QNT-5, QNT-Y (10 µg/mL each). (*) p<0.05; (**) p<0.001 Kruskal-Wallis test with Dunn's Multiple Comparison Test. Mean with SEM bars are shown.

T cell responses elicited by T1BT* and T1BT*-Y were assessed in splenocytes of DR4-transgenic mice using IFN-γ as a readout. IFN-γ responses were measured *in vitro* after stimulation with various assay antigens (T1BT*, T1BT*-Y, T*-1; QNT-5, QNT-Y, T1 and HA_306–318_) and compared to splenocytes from mock-immunized animals. The experimental protocol is shown in figure 7A, and figure 7B summarizes the results observed after 2^nd^ dose (red symbols) and 3^rd^ dose (gray symbols) immunization. In general the observed responses were considerably weaker than those previously reported in non-transgenic mice using a similar immunization and assay strategy [59]. The responses in T1BT* (diamonds) and T1BT*-Y (circles) immunized mice to their respective immunogens were significantly higher than those in control mice (squares, immunized with PBS/montanide). Weak responses to QNT-5 and QNT-Y peptides were also observed. IFN-γ responses to T*-1, T1 and the control HA peptide were not significantly higher than those in control mice for either immunogen. Overall no significant difference in the IFN-γ response to any assay antigen was detected in comparisons of mice immunized with T1BT* and T1BT*-Y.

### IFN-γ response to QNT-Y wanes over time and at forty-five days after third dose is weaker than the response to QNT-5

Antibody responses in T1BT*-Y were lower over time when compared with T1BT* immunized mice (Figure 5C). To evaluate if the IFN-γ T cells response to QNT-5 and QNT-Y followed a similar trend, splenocytes were obtained from mice immunized with T1BT* and T1BT*Y 45 days after the third immunization dose (Figure 7A) and their IFN-γ response determined as above. We found that similarly to antibody responses, IFN-γ responses at 45 days to both QNT-5 and QNT-Y were significantly greater in mice vaccinated with T1BT* as compared to T1BT*-Y (Figure 7C). Moreover responses in T1BT*-Y mice were not significant as compared to those in control mice. These results suggest that QNT-5 is more effective than QNT-Y in inducing long-term IFN-γ T cells.

### Human CD4 T cells primed in vitro with QNT-5 exhibit a central memory phenotype and are cross reactive with QNT-Y

**Figure 8:**
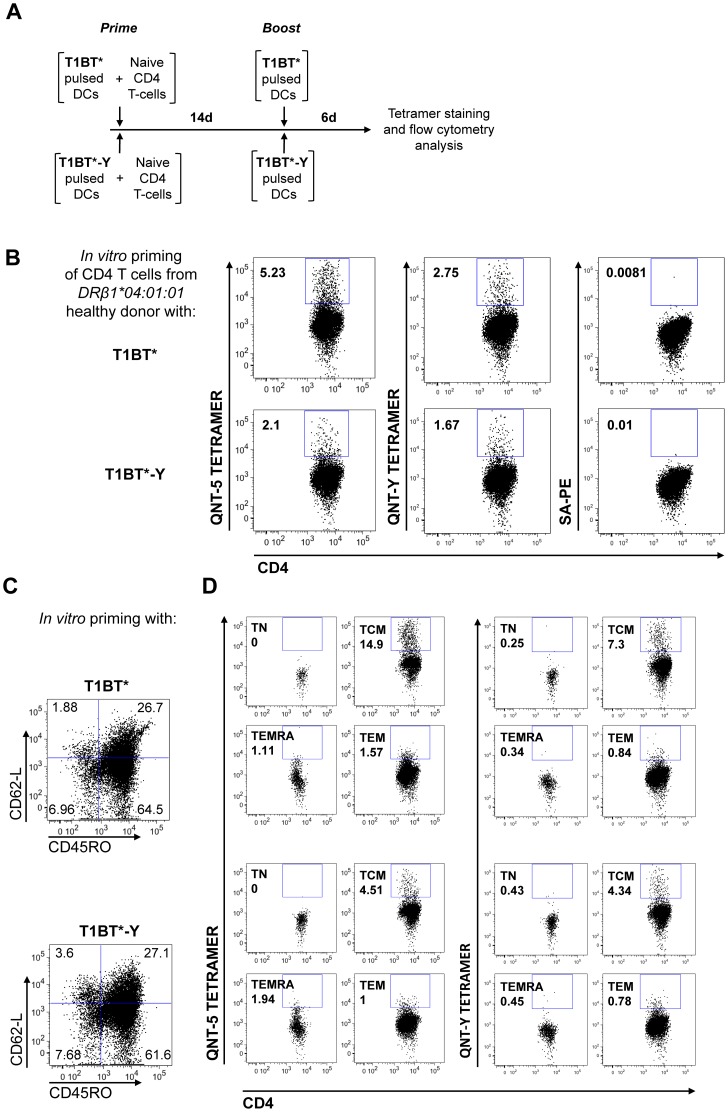
Human CD4 T cells specific for the QNT-5 epitope cross-react with QNT-Y and vice versa. (A) Prime-boost scheme used for the *in vitro* priming of naïve CD4 T cells with T1BT* or T1BT*-Y pulsed DCs from a DRβ1*04:01:01 healthy individual. (B) Flow cytometric analysis of CD4 naïve T cells following co-culture for 14 days with DCs pulsed with T1BT* (top) or T1BT*-Y (bottom) and re-stimulated with peptide-pulsed DCs for 6 days. Plots show the percentages of CD4 T cells stained with SA-PE QNT-5, with SA-PE QNT-Y tetramers or with SA-PE only among CD3+CD4+ T cells after 21 days in culture. (C) CD4+ naïve (TN) (CD45RO- CD62-L+), central memory (TCM) (CD45RO+ CD62-L+), effector (TEM) (CD45RO+ CD62-L-) and terminal effector (TEMRA) (CD45RO- CD62-L-) T cell sub-populations were identified using flow cytometry by staining the cells after 21 days in culture with fluorescently labeled anti-CD45RO and anti-CD62-L antibodies. The numbers in the plots correspond to the percentage of each sub-population among CD3+CD4+ double-positive T cells. (D) Plots showing the percentages of CD4 T cells positive for QNT-5 and QNT-Y tetramers present among the TN, TCM, TEM or TEMRA T cell sub-populations after 21 days in culture.

Successful vaccination relies in the generation of long-term memory T cells. The higher long-term Ab and IFN-γ cellular responses induced by the T1BT* construct as compared to T1BT*-Y suggests that QNT-5 fosters the generation of central memory T cells more efficiently than QNT-Y despite the improved HLA-DR4 binding of QNT-Y. To investigate this in a human setting, we looked at the ability of T1BT* and T1BT*-Y to prime naïve CD4 T cells *ex vivo*. DCs were pulsed with T1BT* or T1BT*-Y and incubated together with naïve CD4 T cells, and three weeks later the cells were stained with anti-CD3, anti-CD4, anti-CD62L, anti-CD45RO antibodies and with DR4 fluorescent tetramers specific for T*-1; QNT-5 and QNT-Y (Figure 8A). The use of fluorescent tetramers allowed us to compare the percentage of CD4 T cells responding to each epitope as well as the percentage of responding cells in memory and effector compartments. DR4/QNT-5 and DR4/QNT-Y tetramer-positive cells were detected in cultures that had been primed with either T1BT* or T1BT*-Y (Figure 8B). T cells elicited against QNT-5 cross-reacted with QNT-Y and vice versa (Figure 8B). The priming of naïve CD4 T cells with T1BT* led into a more vigorous expansion of central memory CD4 T cells specific for QNT-5 than observed for QNT-Y in cells primed with T1BT*-Y (14.9% vs. 4.51% of TCM respectively in figure 8D and table 4). The percentages of effector CD4 T cells specific for each epitope (TEF or TEM in table 4), were not remarkably different between cultures primed with either T1BT* or T1BT*-Y.

**Table 4:** Percentage of tetramer positive CD4 T cells in different CD4 T cell sub-populations.

Cell line	Tetramer	% TN	% TCM	% TEF	% TEM
T1BT*	T*-1	0.76%	7.68%	0.87%	1.99%
	QNT-5	0.00%	**14.9%**	1.1%	1.57%
	QNT-Y	0.25%	7.3%	0.34%	0.84%
	SA-PE	0.00%	0.06%	0.00%	0.00%
T1BT*-Y	T*-1	0.00%	8.19%	0.79%	1.17%
	QNT-5	0.18%	**4.51%**	1.94%	1.0%
	QNT-Y	0.43%	4.34%	0.45%	0.78%
	SA-PE	0.00%	0.06%	0.00%	0.00%

## Discussion

In spite of significant advances in the understanding of the biology of *Plasmodium* parasites and the immune response elicited by these pathogens, there is not yet a subunit vaccine capable of eliciting long lasting protection. Multiple studies have demonstrated that antibodies to the repeat units in the CS protein can neutralize *Plasmodium* sporozoites [13], [60]–[63]. In humans, although protected individuals have higher anti-repeat antibody titers than non-protected individuals, the correlation between protection and anti-CS antibody titers is not perfect (reviewed in [9], [64]). A major limitation for a *P. falciparum* vaccine based on antibody responses is that the CS repeats region does not elicit T cell responses in most individuals [18], [19]. The search for CD4 T cell epitopes in the *P. falciparum* CS protein has resulted in the identification of 4 T cell epitopes in the C-terminal region of this protein designated as a T*, Th2R, Th3R and CS.T3 [18], [30], [33]. T cell responses to Th2R, Th3R and T* have also been reported in vaccines immunized with the malaria vaccine RTS,S [35]. Genetic variability in *P. falciparum* CS regions that harbor Th2R and Th3R [18], [55], [65] is a major consideration in vaccines engineered with these T helper epitopes [54], [66], [67]. However, it is not clear if polymorphism is a consequence of immunological pressure [55], [68].

In this work we studied a highly conserved C-terminal epitope from T* designated as QNT-5. Contrary to other major CD4 T cell epitopes (T*-1, Th2R and Th3R) [18], [30], [39], QNT-5 spans a highly conserved sequence and for this reason is a very attractive epitope for subunit vaccine development. We identified a DR4-binding register in QNT-5 characterized primarily by pocket 1 (L_335_) and pocket 6 (S_340_) anchor residues with smaller contributions from pocket 4 (E_338_) and pocket 7 (P_341_). Measurement of different biophysical parameters of this MHCII-peptide interaction revealed the highly unstable character of DR4/QNT-5 complex in the presence of HLA-DM. As a step toward the development of a strategy for stabilizing this MHC-peptide interaction in the presence of HLA-DM, we investigated the role of the pocket 1 (P1) side chain, thought to be the major determinant of HLA-DR binding. An MHCII-peptide complex stable in the presence of HLA-DM was achieved after improving the binding capacity of QNT-5 to HLA-DR4 by replacing the naturally occupying L_335_ residue at P1 with the optimal P1 side chain tyrosine. Previous studies of some peptide-based vaccines have enhanced immunogenicity by improving MHC-peptide interactions [69]–[71]. The *in vitro* peptide binding results reported here suggested that this approach might potentially provide a possible strategy to improve the immunogenicity of malaria vaccine candidates based on T*. However, *in vivo* studies in DR4 transgenic animals indicated that any improvement in immunogenicity due to stabilizing the MHC-peptide interaction was modest and transient, and the unstable parent QNT-5 epitope elicited better long-term immune responses than the stable modified QNT-Y.

Our in vitro peptide binding studies relied on inhibition of binding of an indicator peptide to MHC DR4 molecules by unlabeled competitor peptides, an assay commonly used to evaluate MHCII-peptide interaction [72]. Although inhibition assays can provide reliable rank measures of relative affinities, the IC_50_ values are not necessarily linear with *K_d_* values. Also, *K_d_* values refer to reactions at equilibrium, but the MHCII-peptide interaction has several steps with very slow kinetics and equilibrium is difficult or impossible to achieve. These reasons led McConnell and colleagues [72], [73] to propose that study of complex kinetic stability with labeled peptides may be advantageous over IC_50_ or *K_d_* values. Moreover, abundant experimental evidence indicates that MHCII-peptide complex stability is highly influenced by HLA-DM molecules [25], [74], [75]. Thus we conducted experiments to assess the stability of DR4/T*-1 and DR4/QNT-5 complexes in the presence and absence of HLA-DM. These experiments showed that while the apparent *K_d_* values of the two peptide complexes were not remarkably affected by HLA-DM (Table 2), the half-lives were significantly lower than those formed by a control viral epitope (DR4/HA), and notably, that DR4/QNT-5 complexes were highly unstable even in absence of HLA-DM.

The issue of peptide editing by HLA-DM has stimulated considerable research in identifying characteristics of class II-peptide complex that can influence the complex susceptibility to HLA-DM. Structural features, including peptide length [76], destabilizing amino-acid residues [77], [78], the rigidity of the P1 pocket of the class II molecules [79], and both anchor interactions and hydrogen bonding [77], [80]–[82] all have been shown to influence class II-peptide complex susceptibility to HLA-DM. In contrast with the limited availability until recently of structural data on HLA-DM's interaction with class II MHC proteins [83]–[85], functional experimental data pointing out the important role of anchor residues in conferring class II-peptide stability to HLA-DM have been published [86], [87]. Lazarski et al. and others show that dissociation kinetics in live cells remarkably influence immunogenicity of peptide-MHC complex *in vivo* [22], [24], [25] and that replacing non-optimal residues at anchor positions is a suitable way to gain resistance to HLA-DM and increase immunogenicity *in vivo*. In their work, poorly immunogenic epitopes can be converted to highly immunogenic epitopes *in vivo* by optimizing peptide anchor residues such as P4 and P6 that foster class II-peptide kinetic stability [25]. Similarly, Rinderknecht et al., observed increased HLA-DM resistance after substitutions in the P4 and P6 pockets [88]. By optimizing the P1 anchor residue we were able to convert DR4/QNT-5 into a stable complex. This could be interpreted as the action of HLA-DM in QNT-5 editing being oriented toward the P1 pocket as proposed by others [77], [82], [85], [89], [90], but whether or not the increased stability to HLA-DM of QNT-Y could be attributed to synergistic effect on QNT-Y of anchor residues beyond P1 such as P4, P6 and P7 as suggested by Lazarski et al [24], [25] and Rinderknecht et al [88] is a possibility that deserve further investigation.

To assess the relative immunogenicity of the parent QNT-5 and P1-optimized QNT-Y epitopes, DR4 transgenic mice were immunized with linear peptides T1BT* or T1BT*-Y, and CD4 T cell IFN-γ responses and anti-CS antibody responses were studied. The T1BT* linear peptide was selected for these studies because *(i)* this subunit vaccine candidate can be produced as a synthetic linear peptide at low cost *(ii)* the T1BT* sequence has already been used in clinical trials and shown to induce relative high anti-sporozoite titers and T cell responses in individuals of multiple MHC haplotypes [36], [38] and *(iii)* in a *P. berghei* transgenic model that expresses the *P. falciparum* repeats T1BT* has been shown to elicit protective immune responses [59], [91]. We expected that the P1 substitution would increase the immunogenicity of T1BT*-Y over T1BT*, as MHCII-peptide stability was dramatically improved by this substitution (Figure 4). Surprisingly, although short-term CD4 T cell and antibody responses in fact were modestly improved by the substitution (Figure 7A and Figure 5C) the improvement was short-lived, and by day 85, forty five days after the last immunization dose, CD4 T cells responding to QNT-Y were detected in numbers significantly lower compared to cells specific for QNT-5 (Figure 7C). The effectiveness of QNT-5 over QNT-Y in long term helper T cell function was also indicated by high anti-(NANP)_6_ titers that lasted up to three months in sera of mice vaccinated with QNT-5 not detected in mice immunized with QNT-Y (Figure 5C). Similarly we observed a higher capacity of DR4/QNT-5 over DR4/QNT-Y to prime human naïve CD4 T cells *in vitro* (Figure 8).

We performed an analysis of IgG isotype responses to the (NANP)_6_ repeat peptide as a surrogate measure of the production of Th2 associated cytokines that provide CD4 help to these responses. Previous studies have reported that inbreed mice immunized with T1BT* constructs have in their serum IgG subtypes associated with both Th1 and Th2 cellular responses [59], [91]. We found that DR4 transgenic mice immunized with T1BT*-Y initially have respectively stronger Th2–associated IgG1 and Th1-associated IgG2/b response than T1BT* immunized mice. However, following a third dose, both IgG1 and IgG2/b are more prominent in T1BT* immunized mice. The Th1-associated IgG2a/b responses in T1BT*-Y mice show a dramatic decrease over time (Figure 6), like the IFN-γ responses observed for this immunogen (Figure 7).

The poor immunogenicity of the stable DR4/QNT-Y complexes is in contrast with previous studies from Sant and coworkers where kinetic stability of MHC-peptide complexes correlates directly with immunodominance during an immune response [22], [24], [25], and with a recent study of Yin et al. [21], where DM-mediated dissociation lifetime and DM-susceptibility values correlated with human CD4 T cell response to peptides from the major core protein A10L of vaccinia virus. The DM-dependent half-life of DR4/QNT-5 was 105 min, three fold lower than the lowest value of any T cell epitope found by Yin et al (∼370 min), indicating an unstable complex that predicts a limited immunogenicity for CD4 T cells. The DM-mediated dissociation lifetime was greatly improved by substitution of the P1 residue in the DR4/QNT-Y complex, as was the intrinsic MHC-peptide dissociation lifetime (Table 2). However, despite the instability of the native DR4/QNT-5 complexes they were able to sustain a long-term IFN-γ responses and high anti-(NANP)_6_ antibody titers, which were not substantially improved by increasing MHC-peptide stability. These results do not fit with the expected relationship between T cell immunogenicity and MHC-peptide stability and particularly resistance to DM-mediated editing.

Why QNT-5 is more effective than QNT-Y in inducing long-term T cell and antibody responses currently is not clear, but at least two possibilities can be envisioned: First, the EWSPCSVTC sequence in QNT-5 has a high degree of homology with thrombospondin type-1 repeats (TSR-1) present in at least 41 human proteins [92], [93], including thrombospondin-1, properdin and F-spondin extracellular matrix proteins [94], [95]. Alignment of predicted DR4-binding epitopes from these self-proteins reveals considerable similarity with QNT-5 and QNT-Y at MHC pocket position P4 and P6 and potential T cell contact residues in position 5 and positions 8 to 12 (Table 5). Thus, T cells that prefer the C-terminus region of T* might be modulated to avoid autoimmune response, for example by Treg-based mechanisms, with the improved stability of QNT-Y resulting in increased modulation. Second, we are using a transgenic mouse model in which antigenic peptides are presented by a hybrid mouse/DR4 molecule [96] that might not have an optimal interaction with mouse DM molecule.

**Table 5:** Homology of the *P. falciparum* CS protein sequence in QNT-5 (strain NF54) and selected self-proteins.

Protein	Sequence ID	Sequence$
CS T*	sp|P19597.2|CSP_PLAFO	QNSLSTEWSPCSVTC
CS T*-Y		---Y-----------
Thrombospondin human	gb|AAK34948.1|AF251058_1	PKW-AQ-----T---
Properdin	gb|AAB63279.1|	RW—WST-A------
VSGP/F-spondin	dbj|BAB18461.1|	LL-PWS---D----G

$ Underlined are MHC anchor residues in P1, P4, and P6; dashes indicate identical residues.

Taken together, our results indicate that the highly conserved QNT-5 Th epitope of T* can be improved for interaction with MHC DRβ1*04:01 molecules and for resistance to HLA-DM editing by inclusion of an optimal P1 residue, and that such epitope engineering can transiently improve IFN-γ and B cell responses. However, the improvement in immunogenicity was short-lived, and long-term immune responses were attenuated by the modification. From our findings it is clear that additional research is needed to understand the factors that govern immune responses to T*.

## Supporting Information

Figure S1 **Direct peptide-binding assays and calculation of apparent binding affinity (** ***K_d_*** **).** (A) Amino-acid sequence and location of T*-1 and QNT-5 epitopes in T*. (B) Plots showing direct binding profile to DR4 of HA, T*, T*-1 and QNT-5 biotin labeled peptides upon binding reactions set up with or without 1 µM of HLA-DM (closed and open symbols respectively). The biotin labeled peptide/DR4 complexes carried out in duplicates was revealed using DR-ELISA; the values plotted as fraction bound are normalized respect to maximum binding achieved by 72 hours. The figure shows one representative experiment out of three performed. Range of *K_d_* values found in reactions performed with and without HLA-DM are shown in table 2.
(TIF)

Table S1 **Relative IC50 values of Alanine substitution analogues and truncated versions of QNT-5.**
(DOCX)
