# Correction: An Unstable Th Epitope of *P. falciparum* Fosters Central Memory T Cells and Anti-CS Antibody Responses

**DOI:** 10.1371/journal.pone.0113028

**Published:** 2014-11-04

**Authors:** The PLOS ONE Staff

Dr. Liusong Yin should be included in the author byline instead of the Acknowledgements. He should be listed as the third author, and his affiliation is 5: the University of Massachusetts Medical School, Department of Pathology and Biochemistry and the Department of Molecular Pharmacology, Worcester, Massachusetts, United States of America. The contributions of this author are as follows: Performed the experiments, analyzed the data.

The correct citation is: Parra-López CA, Bernal-Estévez D, Yin L, Vargas LE, Pulido-Calixto C, et al. (2014) An Unstable Th Epitope of *P. falciparum* Fosters Central Memory T Cells and Anti-CS Antibody Responses. PLoS ONE 9(7): e100639. doi:10.1371/journal.pone.0100639

The Acknowledgements should read: We thank Dr. Alberto Moreno for providing (NANP)6 peptide. DBE is very thankful to Dr. Fabio Méndez Rivera and Fundación Salud de los Andes for their support. We express our gratitude to blood donors and to Dr. Bernardo Camacho at the Hemocentro Distrital. We finally thank J. Polli, C. Szklarz and K. Graslie from the UMASS animal facility.
