# Supplementary figures and images for: An Unstable Th Epitope of P. falciparum Fosters Central Memory T Cells and Anti-CS Antibody Responses
Source: PLoS One. 2014 Jul 1;9(7):e100639. doi: 10.1371/journal.pone.0100639 (PMC4077652; doi:10.1371/journal.pone.0100639)

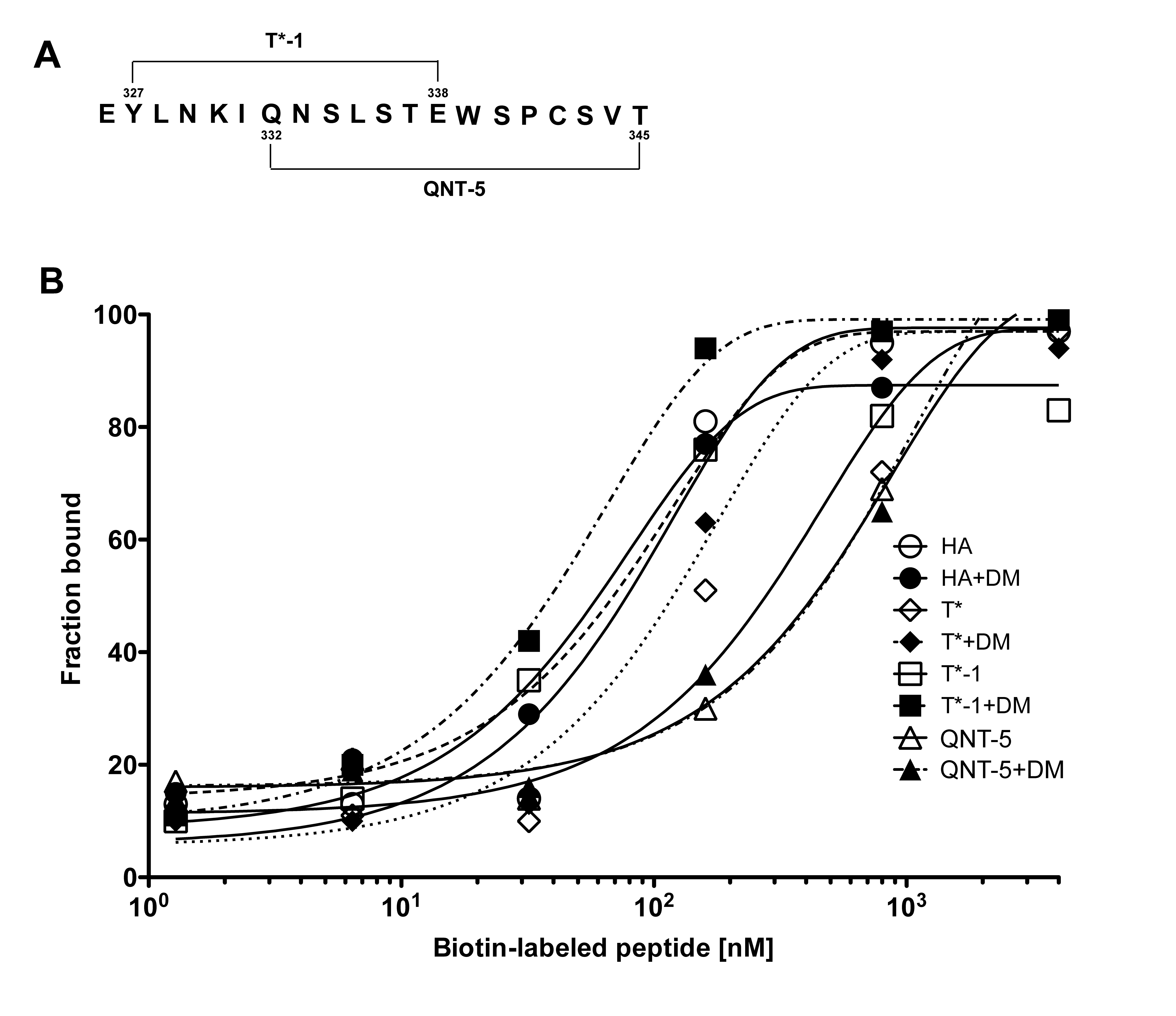

Supplement: Figure S1 — Direct peptide-binding assays and calculation of apparent binding affinity ( Kd ). (A) Amino-acid sequence and location of T*-1 and QNT-5 epitopes in T*. (B) Plots showing direct binding profile to DR4 of HA, T*, T*-1 and QNT-5 biotin labeled peptides upon binding reactions set up with or without 1 µM of HLA-DM (closed and open symbols respectively). The biotin labeled peptide/DR4 complexes carried out in duplicates was revealed using DR-ELISA; the values plotted as fraction bound are normalized respect to maximum binding achieved by 72 hours. The figure shows one representative experiment out of three performed. Range of Kd values found in reactions performed with and without HLA-DM are shown in table 2. (TIF) [file pone.0100639.s001.tif]
