# Supplementary material for: An Unstable Th Epitope of P. falciparum Fosters Central Memory T Cells and Anti-CS Antibody Responses
Source: PLoS One. 2014 Jul 1;9(7):e100639. doi: 10.1371/journal.pone.0100639 (PMC4077652; doi:10.1371/journal.pone.0100639)
Supplement: Table S1 — Relative IC50 values of Alanine substitution analogues and truncated versions of QNT-5. (DOCX) [file pone.0100639.s002.docx]

**Table S1. Relative IC50 values of Alanine substitution analogues and truncated versions of QNT-5**

| **Peptide** | **Mean IC50 (µM)** | **SD** |
| --- | --- | --- |
| QN-332-345 | 1.57 | 0.3458 |
| Q332A | 1.19 | 0.2570 |
| N333A | 2.65 | 0.4200 |
| S334A | 1.14 | 0.1970 |
| L335A | 68.43 | 12.8900 |
| S336A | 0.91 | 0.2050 |
| T337A | 1.30 | 0.3380 |
| E338A | 3.40 | 2.2250 |
| W339A | 1.42 | 0.2500 |
| S340A | 24.64 | 0.7510 |
| P341A | 7.75 | 1.1710 |
| C342A | 0.73 | 0.0737 |
| S343A | 0.70 | 0.0709 |
| V344A | 0.44 | 0.0950 |
| T345A | 0.71 | 0.1200 |
| QN-332-343 | 5.58 | 1.036100 |
| QN-334-345 | 2.82 | 0.731800 |
| QN-335-343 | 75.79 | 23.437000 |
| QN-336-343 | 6743.30 | 3544.500000 |
| HA | 0.31 | 0.050332 |
